# Inadequate Antibody Response to Rabies Vaccine in Immunocompromised Patient

**DOI:** 10.3201/eid1809.111833

**Published:** 2012-09

**Authors:** Eran Kopel, Gal Oren, Yechezkel Sidi, Dan David

**Affiliations:** The Chaim Sheba Medical Center, Tel Hashomer, Israel (E. Kopel, G. Oren, Y. Sidi);; Ministry of Health, Tel Aviv, Israel (E. Kopel); Sackler School of Medicine, Tel Aviv University, Tel Aviv (Y. Sidi);; and Kimron Veterinary Institute, Bet Dagan, Israel (D. David)

**Keywords:** rabies, viruses, vaccine, antibody response, failure, immunocompromised, prophylaxis, case report, literature review, post-exposure, pre-exposure, registry, Rhabdoviridae, Lyssavirus

## Abstract

We describe an inadequate antibody response to rabies vaccine in an immunocompromised patient. A literature search revealed 15 additional immunocompromised patients, of whom 7 did not exhibit the minimum acceptable level of antibodies after a complete postexposure prophylaxis regimen. An international rabies registry is needed to provide a basis for determining appropriate vaccination protocols.

Rabies is a rapidly progressive viral encephalitis caused by RNA viruses of the family *Rhabdoviridae*, genus *Lyssavirus*. Dogs are the major reservoir for these viruses worldwide and usually transmit the virus by conveying their infected saliva through the penetrated skin of bitten humans or animals. The usual incubation period in humans ranges from 10 days to 1 year (average 20–60 days). Rabies causes 30,000–70,000 human deaths throughout the world each year. The rabies-related death rate is ≈100% in unvaccinated patients. Thus, preexposure prophylaxis and postexposure prophylaxis (PEP) are the main effective approaches for treating the disease (*1**–**3*).

We describe a case in which an acceptable antibody response to rabies vaccine did not develop in an immunocompromised patient. We also searched the literature for similar cases and summarize the demographic, clinical, and epidemiologic characteristics of such case-patients to date.

## The Patient

A 74-year-old woman was hospitalized at the Chaim Sheba Medical Center in August 2011; she reported progressive general weakness that had begun several months before her admission. Her blood count on admission showed severe lymphopenia (250 lymphocytes/µL). In addition, her recent medical history suggested that she had experienced a category II or III exposure (*4*) to a monkey’s bite, as classified by the World Health Organization (WHO), 10 days before admission, while she was traveling in a country where rabies was endemic.

The patient was treated with the standard PEP regimen for immunocompromised patients in accordance with Israel Ministry of Health guidelines at the time she was admitted (*5*). These guidelines also corresponded to the latest guidelines of WHO and of the American Advisory Committee on Immunization Practices (ACIP) regarding PEP for immunocompromised patients (*4**,**6*). In brief, 5 doses of cell culture rabies vaccine, of which both purified Vero cell vaccine (PVRV) and purified chick embryo cell vaccine are available in Israel, are administered intramuscularly on days 0 (together with 20 IU/kg of human rabies immune globulin), 3, 7, 14, and 28.

The PEP regimen for the patient began 12 days after her potential exposure to rabies virus through the monkey bite with the administration of the PVRV vaccine (Verorab, batch E1036; Sanofi Pasteur SA, Lyon, France). On day 15 of the PEP regimen, 2 vials of serum and 1 vial of cerebrospinal fluid (CSF), each of which contained >2 mL of fluid from routine samples, were tested for rabies virus neutralizing antibodies (VNA) by the National Rabies Laboratory at Kimron Veterinary Institute. These samples were adequately cooled until the time of analysis. VNA titers were measured by using the rapid fluorescent focus inhibition test (*7*).

No detectable levels of VNA were measured either in CSF (<0.04 IU/mL) or in the serum samples (<0.07 IU/mL in both vials). The acceptable WHO cut-off level, indicating an adequate adaptive immune response, is 0.5 IU/mL (*4*); the ACIP cut-off level is 0.1 IU/mL (complete virus neutralization at serum dilution of 1:5) (*6*).

Before the fifth PVRV could be administered, the patient died of sepsis, most likely of nosocomial origin, induced by her rapidly progressive immunodeficient condition. The pathologic and histologic findings from a lymph node biopsy specimen were concordant with the diagnosis of advanced B-cell lymphoma.

Because of the challenging clinical conditions that we encountered (an immunocompromised patient in need of rabies PEP without an apparent adequate adaptive immune response to the standard regimen), we searched the medical literature for similar reported cases to describe more completely, and in proper context, the epidemiologic and public health issues that were evoked by our case-patient. We conducted a search in the MEDLINE database using the PUBMED website (http://www.ncbi.nlm.nih.gov/pubmed) on September 15, 2011. We used various combinations of the following search terms or Medical Subject Heading terms: “rabies,” “vaccine,” “failure,” “immune response,” “human,” and “immunocompromised host.”

By this strategy, we found 5 publications (*8**–**12*), which reported 15 immunocompromised patients who were possibly exposed to rabies and were given a PEP regimen (Table). Various underlying illnesses were responsible for the immunodeficiency states of these patients. Eight patients had AIDS (*8**,**10*), defined as laboratory confirmation of HIV infection and CD4+ T-lymphocyte count of <200 cells/µL for patients >13 years of age or if the criteria for HIV infection were met and at least 1 of the AIDS-defining conditions had been documented for patients 18 months to <13 years of age (*13*). Five patients were infected with HIV, of whom >1 patients had AIDS, but this information was not further specified in the original publication (*9*). One patient had high-grade B-cell lymphoma (*11*), and 1 patient had received a kidney transplant (*12*).

**Table:** Summary of published reports on inadequate antibody response to rabies vaccine in immunocompromised patients*

Characteristic	Case-patient 1 (*8*)	Case-patients 2–6 (*9*)	Case-patients 7–13 (*10*)	Case-patient 14 (*11*)	Case-patient 15 (*12*)	Case-patient in this study
Age, y	6	NA	7–38	55	55	74
Sex	F	NA	4 F, 3 M	M	M	F
Country	Thailand	Thailand	Thailand	Israel	Mexico	Israel
Vaccination year	1998	NA	1998–1999	1999	2009	2011
Underlying illness	AIDS	HIV infection	AIDS	Advanced B-cell lymphoma	Kidney transplant recipient	Advanced B-cell lymphoma
Leukocyte count at baseline	44 CD4^+^/μL	111–250 CD4^+^/μL	25–199 CD4^+^/μL	NA	NA	250 lymphocytes/μL
Vaccine type (dose)	PVRV (0.1 mL)	PVRV (0.1 mL)	PVRV (0.1 mL)	PCECV (1.0 mL)	PVRV (0.5 mL)	PVRV (0.5 mL)
Standard PEP regimen (d)	4 sites, ID (0, 3, 7); 2 sites, ID (60, 90)	2 sites, ID (0, 3, 7); 1 site, ID (28, 90)	4 sites, ID (0, 3, 7); 2 sites, ID (60, 90)	1 site, IM (0, 3, 7, 14, 28)	1 site, IM (0, 3, 7, 14, 28)	1 site, IM (0, 3, 7, 14)†
Ig at day 0 (dose)	NA	Equine rabies Ig (40 IU/kg)	Human rabies Ig (20 IU/kg)	Human rabies Ig (20 IU/kg)	Human rabies Ig (20 IU/kg)	Human rabies Ig (20 IU/kg)
VNA titer in case-patients without adequate vaccine response (d of last measurement)	<0.07 IU/mL in serum (90)	Undetectable and <0.5 IU/mL in serum samples for 2 patients (90)	<0.04 IU/mL and 0.23 IU/mL in samples for 2 patients (90)	0.2 IU/mL in serum (30)	0.31 IU/mL in serum (28)	<0.07 IU/mL in serum and <0.04 IU/mL in CSF (15)

*NA, not available; PVRV, purified Vero cell vaccine; PCECV, purified chick embryo cell vaccine; PEP, postexposure prophylaxis; ID, intradermal; IM, intramuscular; VNA, rabies virus neutralizing antibodies; CSF, cerebrospinal fluid. †The patient died before the scheduled fifth dose on day 28.

Of these 15 patients, 7 did not show the acceptable WHO cut-off VNA titer level at any of the reported measurement points during and after administration of the initial PEP regimen. Whether an adequate immune response had eventually developed in a patient due to any additional vaccine doses beyond the original PEP protocol was not accounted for in our literature summary.

## Conclusions

We report a patient who had an inadequate rabies antibody response to the standard PEP regime probably due to an underlying immunodeficiency condition. Besides this case-patient, from a survey of the literature, we found reports of an additional 7 immunocompromised patients who also demonstrated a lack of acceptable response level. These patients had followed various rabies PEP regimens with different vaccine types, administration methods, number of injection sites, and doses. However, despite those measures, an adequate immune response did not develop in these 7 patients during the entire follow-up period of each study.

The WHO cut-off titer level of 0.5 IU/mL (*4*), an equivalent to complete virus neutralization at a serum dilution of ≈1:50 by the rapid fluorescent focus inhibition test, as well as the lower titer recommend by ACIP (serum dilution of 1:5) (*6*) are arbitrary laboratory values that do not correlate directly with seroprotection. Moreover, the WHO cut-off titer was originally based on the adequate immune response levels required when repeatedly monitoring healthy patients who needed a prophylactic preexposure regime on a regular basis, for example, veterinarians (*14*).

The VNA titer response ideally should be determined 2–4 weeks (WHO) or 1–2 weeks (ACIP) after the last dose of vaccine to assess whether an additional dose is needed (*4**,**6*). This points to a limitation of the current study, which had 1 measurement point on day 15; thus, the measurement time in our study might merely reflect an immune response to the first 3 doses (days 0, 3, and 7). The same limitation also applies to the previously published studies (*8**–**12*), all of which had the last VNA titer measurement on the same day or only a few days after the end of the vaccination regimen. Nevertheless, not even a slight increase in the VNA titers was observed on day 15 in either the CSF or serum samples, which could possibly imply a further lack of immune response. Low or undetectable VNA levels on day 90, the last day of the PEP regimen, were similarly observed in some of the previous studies (*8**–**10*). Thus, we could not expect the antibody titer to rise much further, even if additional measurements would have been taken 1–4 weeks after the last vaccine dose as indicated by the guidelines.

In conclusion, current epidemiologic knowledge and existing PEP regimens might not provide enough reassurance for public health experts and attending clinicians when advising and treating immunocompromised patients. Establishing a collaborative international rabies registry with a particular emphasis on immunocompromised patients could therefore provide evidence that would contribute to decisions regarding the appropriate vaccination protocol.

## References

[1] Rupprecht CE, Gibbons RV. Clinical practice. Prophylaxis against rabies. N Engl J Med. 2004;351:2626–35. 10.1056/NEJMcp042140 15602023

[2] Hankins DG, Rosekrans JA. Overview, prevention, and treatment of rabies. Mayo Clin Proc. 2004;79:671–6. 10.4065/79.5.671 15132411

[3] Warrell MJ, Warrell DA. Rabies and other lyssavirus diseases. Lancet. 2004;363:959–69. 10.1016/S0140-6736(04)15792-9 15043965

[4] World Health Organization. Rabies vaccines: WHO position paper. Wkly Epidemiol Rec. 2010;85:309–20 [cited 2011 Nov 13]. http://www.who.int/wer/2010/wer8532.pdf

[5] Israel Ministry of Health, Public Health Services. Guidelines for rabies prevention [in Hebrew]. Circular no. 3/2010, May 26, 2010.

[6] Rupprecht CE, Briggs D, Brown CM, Franka R, Katz SL, Kerr HD, Use of a reduced (4-dose) vaccine schedule for postexposure prophylaxis to prevent human rabies: recommendations of the advisory committee on immunization practices. MMWR Recomm Rep. 2010;59:1–9. 20300058

[7] Smith JS, Yager PA, Baer GM. A rapid reproducible test for determining rabies neutralizing antibody. Bull World Health Organ. 1973;48:535–41. 4544144 PMC2482941

[8] Pancharoen C, Thisyakorn U, Tantawichien T, Jaijaroensup W, Khawplod P, Wilde H. Failure of pre- and postexposure rabies vaccinations in a child infected with HIV. Scand J Infect Dis. 2001;33:390–1. 10.1080/003655401750174183 11440231

[9] Jaijaroensup W, Tantawichien T, Khawplod P, Tepsumethanon S, Wilde H. Postexposure rabies vaccination in patients infected with human immunodeficiency virus. Clin Infect Dis. 1999;28:913–4. 10.1086/517241 10825063

[10] Tantawichien T, Jaijaroensup W, Khawplod P, Sitprija V. Failure of multiple-site intradermal postexposure rabies vaccination in patients with human immunodeficiency virus with low CD4+ T lymphocyte counts. Clin Infect Dis. 2001;33:e122–4. 10.1086/324087 11641838

[11] Hay E, Derazon H, Bukish N, Scharf S, Rishpon S. Postexposure rabies prophylaxis in a patient with lymphoma. JAMA. 2001;285:166–7. 10.1001/jama.285.2.166 11176806

[12] Rodríguez-Romo R, Morales-Buenrostro LE, Lecuona L, Escalante-Santillán N, Velasco-Villa A, Kuzmin I, Immune response after rabies vaccine in a kidney transplant recipient. Transpl Infect Dis. 2011;13:492–5. 10.1111/j.1399-3062.2011.00665.x 21883758 PMC7564869

[13] Schneider E, Whitmore S, Glynn KM, Dominguez K, Mitsch A, McKenna MT; Centers for Disease Control and Prevention. Revised surveillance case definitions for HIV infection among adults, adolescents, and children aged <18 months and for HIV infection and AIDS among children aged 18 months to <13 years—United States, 2008. MMWR Recomm Rep. 2008;57:1–12. 19052530

[14] World Health Organization. WHO expert consultation on rabies. World Health Organ Tech Rep Ser. 2005;931:1–88. 16485446

